# The Cerebellum Is Sensitive to the Lexical Properties of Words During Spoken Language Comprehension

**DOI:** 10.1162/nol_a_00126

**Published:** 2024-08-15

**Authors:** Hannah Mechtenberg, Christopher C. Heffner, Emily B. Myers, Sara Guediche

**Affiliations:** Department of Psychological Sciences, University of Connecticut, Storrs, CT, USA; Department of Communicative Sciences and Disorders, University at Buffalo, Buffalo, NY, USA; Department of Speech, Language and Hearing Sciences, University of Connecticut, Storrs, CT, USA; College of Science and Mathematics, Augusta University, Augusta, GA, USA

**Keywords:** cerebellum, continuous listening, fMRI, lexical processing, prediction, speech perception

## Abstract

Over the past few decades, research into the function of the cerebellum has expanded far beyond the motor domain. A growing number of studies are probing the role of specific cerebellar subregions, such as Crus I and Crus II, in higher-order cognitive functions including receptive language processing. In the current fMRI study, we show evidence for the cerebellum’s sensitivity to variation in two well-studied psycholinguistic properties of words—lexical frequency and phonological neighborhood density—during passive, continuous listening of a podcast. To determine whether, and how, activity in the cerebellum correlates with these lexical properties, we modeled each word separately using an amplitude-modulated regressor, time-locked to the onset of each word. At the group level, significant effects of both lexical properties landed in expected cerebellar subregions: Crus I and Crus II. The BOLD signal correlated with variation in each lexical property, consistent with both language-specific and domain-general mechanisms. Activation patterns at the individual level also showed that effects of phonological neighborhood and lexical frequency landed in Crus I and Crus II as the most probable sites, though there was activation seen in other lobules (especially for frequency). Although the exact cerebellar mechanisms used during speech and language processing are not yet evident, these findings highlight the cerebellum’s role in word-level processing during continuous listening.

## INTRODUCTION

During spoken language perception, the brain performs a breathtaking array of functions to transform the incoming speech signal into a meaningful message. From segmenting the continuous speech signal into discrete words to assigning each speech stream to the correct talker—all while simultaneously building a coherent narrative—it is no wonder that this process engages interacting brain networks comprising cortical and subcortical structures. Despite evidence that subcortical areas and the cerebellum are recruited for higher-order cognitive processing (Ackermann et al., 2007; Ashida et al., 2019; Guediche et al., 2015; Stoodley & Schmahmann, 2010), neuroanatomical research of speech and language has almost entirely focused on the cerebral cortex (e.g., Hickok & Poeppel, 2007). Although the cerebellum is relatively well studied in the sensorimotor domain (for review, see Ohyama et al., 2003), it remains a neural underdog in the language domain. Due in part to significant developments in understanding the cerebellum’s neural circuitry and its connections to cortical and subcortical areas (Alvarez & Fiez, 2018; Balsters et al., 2013; Bostan et al., 2013; Buckner, 2013; Diedrichsen, 2006; Fiez et al., 1992; Kelly & Strick, 2003; Leiner et al., 1994; Marek et al., 2018; Middleton & Strick, 2000; Ramnani, 2012; Stoodley & Schmahmann, 2009; Xue et al., 2021), and insight into its contributions to the perception and production of language (Argyropoulos, 2016; Booth et al., 2007; Guediche et al., 2015; Kotz & Schwartze, 2010; Manto & Mariën, 2015; Marek et al., 2018; Mariën et al., 2014; Moberget & Ivry, 2019; Pleger & Timmann, 2018; van Dun et al., 2016), interest in the role of the cerebellum in mapping the speech signal to phonetic and lexical units has grown over the past few decades.

The uniform microarchitecture of the cerebellum has led to the hypothesis that a single (or multiple) universal cerebellar mechanism(s) may be adaptable to motor and non-motor domains (Diedrichsen et al., 2019; Ito, 2008), perhaps achieved through unique structural and functional connections with cortex (Booth et al., 2007; Guell et al., 2018; Xue et al., 2021). The cerebellum has a remarkable amount of functional diversity in involvement in non-motor language-related tasks (Desmond et al., 1997; Desmond & Fiez, 1998; D’Mello et al., 2017; Durisko & Fiez, 2010; Moberget & Ivry, 2016; Stoodley et al., 2012; Stoodley & Schmahmann, 2009; van Dun et al., 2016), inspiring calls to determine a potential cerebellar system perhaps comprised of one or multiple adaptable mechanisms that contribute to language processing (Argyropoulos, 2016; Ito, 2005, 2008; Moberget & Ivry, 2016; Skipper & Lametti, 2021; Sokolov et al., 2017). If the cerebellum recruits spatially segregated regions, then characterizing its contributions to higher-order language processes can be informed by precisely defining the specific loci for processing the basic properties of language, including the lexical properties of words.

Although recent work suggests that the cerebellum may be involved in word-level conceptual processing (LeBel et al., 2021), no studies to date have directly measured cerebellar responses to lexical frequency and phonological neighborhood density—two properties known to influence word recognition during speech perception. Examining the cerebellar response to these two fundamental properties of the lexicon allows for the exploration into the potential roles of the cerebellum during the dynamic process of lexical retrieval. One potential mechanism is that the cerebellum encodes and adjusts internal models (Argyropoulos, 2016; E et al., 2014; Ishikawa et al., 2016) which can serve as the basis for generating and adjusting linguistic predictions. Put simply, an internal model is an adaptive, context-specific representation that allows for efficient processing of an incoming sensory experience (Moberget et al., 2014). A critical affordance of internal models, both in the motor and cognitive domains, is the ability to predict what will happen next and to calculate prediction errors that capture the unexpected (Ito, 2008). An increasing number of empirical studies have tested hypotheses related to the cerebellum’s role in linguistic predictions (Guediche et al., 2015; Lesage et al., 2012, 2016; Moberget et al., 2014) prompting a number of review articles on the topic (e.g., Pleger & Timmann, 2018; Skipper & Lametti, 2021).

Another proposed role for the cerebellum concerns semantic integration. Focal disruption of the right posterior cerebellum interrupts judgments on the semantic relatedness of word pairs (Gatti et al., 2020). Further, in studies of continuous listening, the cerebellum is shown to be sensitive to dissociable conceptual feature dimensions of single words (LeBel et al., 2021). Prediction and integration—the proposed cerebellar roles sketched here—are both connected to the dynamic process of lexical retrieval, though we note that the current study is unable to fully distinguish between them during continuous listening. Rather, here, we focus on identifying the spatial loci of cerebellar sensitivity to lexical frequency and phonological neighborhood density during task-free continuous listening; conditions that best approximate natural lexical processing.

Lexical frequency and phonological neighborhood density have shaped theoretical and computational accounts of lexical processing (Benkı´, 2003; Besner & Young, 2022; Brysbaert et al., 2018; Chen & Mirman, 2015; Dahan et al., 2001; Luce & Pisoni, 1998; Savin, 1963). Lexical frequency is a measure of the commonness of a word, while phonological neighborhood density refers to the number of words that differ from a target word by one phoneme—whether added, subtracted, or changed (Vitevitch et al., 1999; Vitevitch & Luce, 2016). The lexical frequency effect describes the impact of frequency on the ease of word recognition. Less frequent words tend to slow access to a word’s form (Dahan et al., 2001). This effect emerges in a variety of active tasks including word naming, lexical decision, and priming (for reviews, see Brysbaert et al., 2011; Brysbaert et al., 2018). Studies of phonological neighborhood density have generally shown that word recognition is slowed for words occupying denser neighborhoods compared to sparser ones (for review, see Vitevitch & Luce, 2016) though this effect is not monotonic (Chen & Mirman, 2015). Beyond recognition, these properties are also likely to affect a word’s integration into the broader context (Cibelli et al., 2015). Taken together, a word’s frequency and number of phonological neighbors likely modulates online word recognition as well as integrative processing.

In the cerebral cortex, word frequency and phonological neighborhood density modulate activity in canonical language regions including the middle temporal gyrus and supramarginal gyrus (Gow, 2012; Peramunage et al., 2011; Prabhakaran et al., 2006; Zhuang et al., 2011). Frontal lobe activation associated with these two properties has also led to domain-general arguments regarding differences in cognitive control demands such as those associated with challenges to lexical selection (Binder et al., 2004; Luthra et al., 2019; Righi et al., 2010; Zhuang et al., 2011). While the effects of frequency and neighborhood density have emerged in the cerebral cortex, of interest is whether activity in the cerebellum is also modulated by lexical-level properties during continuous listening.

The current functional magnetic resonance imaging (fMRI) study was passive in nature; participants only listened to a podcast without any additional task. The vast majority of studies (behavioral and neural) examining lexical frequency and phonological neighborhood density effects have used artificial active tasks that involve making decisions about words presented in isolation (e.g., lexical decision in Dahan et al., 2001); leaving open whether these effects emerge in more ecologically valid contexts, such as when listening to a podcast. Recent studies using fMRI have shown substantial success in modeling semantic, syntactic, and lexical-level properties of continuous speech and text (Brennan, 2016; Brennan et al., 2016; LeBel et al., 2021; Wehbe et al., 2014) despite the sluggish nature of the hemodynamic response in relation to the rapid nature of conversational speech. The potential for fMRI to capture these rapidly occurring lexically modulated effects in the cerebellum is especially appealing as other neuroimaging techniques are not as well suited for capturing responses in such deep-seated structures. Thus, we follow the precedent set by other studies and model words (verbs, nouns, adjectives, adverbs, and function words) using individual, canonical hemodynamic response functions (HRFs) to assess how word-level processing modulates cerebellar activity during continuous listening (Brennan et al., 2016). Critically, we chose an analysis approach—amplitude modulated regression—that allows us to model the relationship between activity in the cerebellum with variation in each lexical property over time. This analysis identifies regions for which by-item differences in the blood oxygen level dependent (BOLD) signal correlates with by-item variation in each lexical property above and beyond activity associated with the time course alone (see Mechtenberg et al., 2021; Xie & Myers, 2018). For example, a positive correlation reflects that for each step that lexical frequency increases, activity in the cerebellum also increases proportionally.

**Figure 1.**
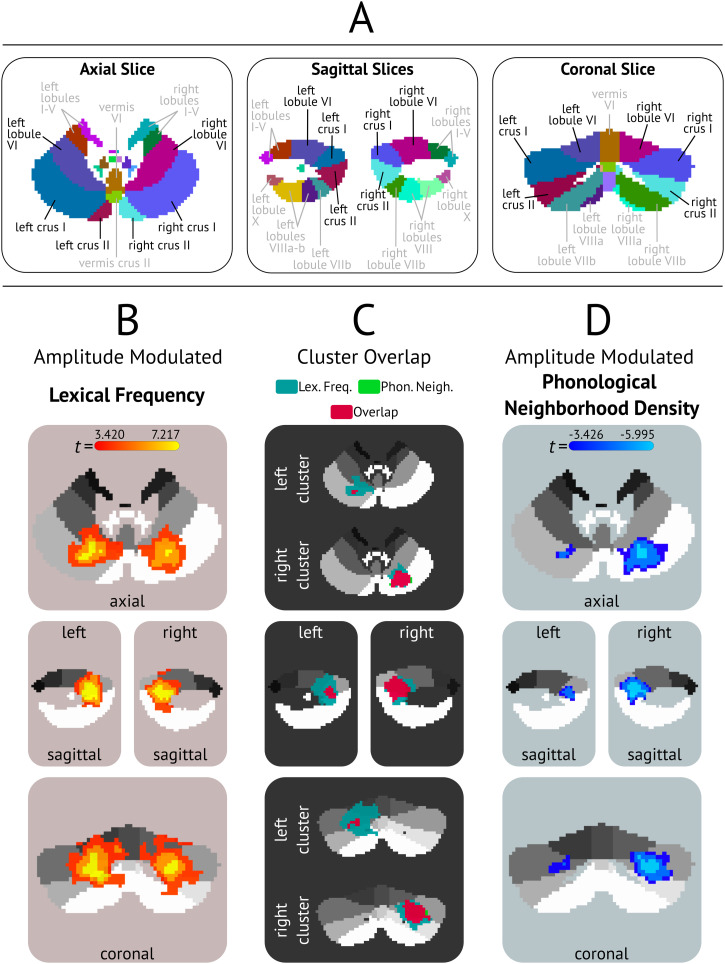
Cerebellar MNI Atlas and group activation maps. (A) MNI cerebellar atlas, labeled with anatomical names. All slice numbers are identical to the slices used to depict the functional results. Slice coordinates are at: axial (z) = 22, left sagittal (x) = 35, right sagittal (x) = 57, coronal (y) = 81. (B) Results of the amplitude-modulated activation for lexical frequency versus implicit baseline, with all regions patterning positively with lexical frequency. Clusters corrected at *p* < 0.01 (voxelwise *p* < 0.001, alpha = 0.01, 32 voxel cluster threshold). (C) Depiction of the amount of overlap of the left and right Crus I/Crus II lexical frequency and phonological neighborhood density clusters. Voxels sensitive to only lexical frequency are shown in teal, voxels sensitive to only phonological neighborhood density are shown in neon green. Overlapping voxels are shown in pink. (D) Results of the amplitude-modulated activation for phonological neighborhood density versus implicit baseline, with activity in all regions negatively correlating with phonological neighborhood density. Clusters corrected at voxelwise *p* < 0.001, alpha = 0.01, 32 voxel cluster threshold).

We hypothesize that the effects of both lexical frequency and phonological neighborhood density should emerge in the postero-lateral cerebellar cortex—Crus I and/or Crus II. Meta-analyses of cerebellar activation across a range of tasks—language, motor, spatial processing, working memory, executive control, and emotion—shows Crus I, Crus II, and Lobule VI (see Figure 1A in Materials and Methods for labeled cerebellar atlas) emerging as the most likely regions to support receptive language processing (Mariën et al., 2014; Pleger & Timmann, 2018; Schmahmann, 2019; Stoodley et al., 2012). Highly relevant to the current paper, activation differences associated with lexical predictability have been observed in Crus I and Crus II. When semantic predictability and sentence-final target-word congruency were systematically manipulated, a region in Crus I/Crus II was activated when final words could be predicted based on context (compared to scrambled sentences) and had even greater activation when final words violated predictions, thought to reflect both predictive processing and cerebellar involvement in error processing (Moberget et al., 2014). The Crus I/Crus II response to lexical prediction also co-occurs with changes in the cortical language network (D’Mello et al., 2017), supporting its contribution (at least in part) to language processes. Further, during continuous listening, Crus I/Crus II (as well as Lobules VIIIA and VIIIB) showed sensitivity to conceptual feature dimensions at the word-level (LeBel et al., 2021).

Despite some confidence in where we expect to find cerebellar sensitivity to lexical frequency and phonological neighborhood density, studies report variability in regards to other subregions—such as Lobule VII, VIII, and IX—that may be recruited for language processing due to additional cognitive demands (e.g., working memory and executive control) that are not a result of processing speech or lexical information specifically (Balsters et al., 2013; Schmahmann, 2019). If effects are observed in these regions, they may reflect differences in domain-general cognitive demands associated with variation in frequency and phonological neighborhood density. Activity in Lobules IV and V (and even portions of Lobule VI; see Figure 1A in Materials and Methods for a labeled cerebellar atlas) has been correlated with language production tasks that engage motor output such as writing and speaking (Manto & Mariën, 2015; Pleger & Timmann, 2018; Schmahmann, 2019; Stoodley et al., 2012). As our “task” is entirely passive and does not require any motor output it would be unexpected, but interesting, if we observed activity in these regions (Galantucci et al., 2006). Finally, it is worth noting that activity in Crus I/Crus II has not exclusively been associated with language-related tasks. For instance, other cognitive functions also reliably activate different subregions of Crus I/Crus II including tasks requiring executive control and working memory (Ashida et al., 2019; Balsters et al., 2013; D’Mello et al., 2020; Habas, 2021). The design of the present study does not allow us to untangle what is language-specific or not during naturalistic listening. As such, our interpretation of potential effects of lexical information will also consider domain-general explanations.

Precise localization of cerebellar effects at the group level can be challenging owing to considerable individual variability in cerebellar anatomy (Diedrichsen, 2006; Kong et al., 2021; Kozlova, 1984). For this reason, in addition to group-level analyses, we also report the results of individual-level analyses, which are spatially aligned to a probabilistic atlas of the cerebellum (Diedrichsen, 2006). The individual-level analyses allow for a more precise spatial localization tailored to each individual. Similar approaches have been used in auditory cortex (see Okada & Hickok, 2006), where variability in the precise location of activity seen at the individual level would have been obscured at the group level. Reporting the individual-level spatial maps also helps to verify that group-level results were not driven by only a few participants.

In the current study, we scanned 79 adults via fMRI while they passively listened to 10 min of a popular podcast. We aim to provide insight into cerebellar involvement in word-level processing by modeling the effects of lexical frequency and phonological neighborhood density during continuous listening at the group and at the individual level. We interpret these findings through both language-specific and domain-general mechanisms.

## MATERIALS AND METHODS

### Participants

Seventy-nine participants (male = 17, female = 61, other = 1) were recruited from the University of Connecticut community as part of a larger project from our group (Heffner & Myers, 2021). All reported that they were native speakers of American English, had no hearing loss or neurological disorders, and were over 18 years of age (*M* = 23.94, range: 18–43). Participants provided written consent based on the University of Connecticut’s Institutional Review Board guidelines and were screened for MRI safety. Each participant was compensated $30/hour.

### Podcast

All participants listened to the same clip of the first 10 min of an episode of National Public Radio’s podcast *Fresh Air With Terry Gross* called “A Science Writer Explores the ‘Perversions and Potential’ of Genetic Tests,” with interviewee Dr. Carl Zimmer (Gross, 2018). The use of this podcast in a research context falls under Fair Use Laws. Please visit https://www.copyright.gov/fair-use/more-info.html for more information. The podcast features a female host and a male interviewee in an interview-style turn-taking discourse. Both talkers spoke in their native language, American English.

Word boundaries were identified by the Penn Forced Aligner (Yuan & Liberman, 2008) and were manually checked for good fit. Word boundaries were adjusted when appropriate. Our two lexical dimensions of interest, lexical frequency (SUBTLWF_US_, Brysbaert & New, 2009) and phonological neighborhood density (complete lexicon, no homophones) were determined for each word within the 10-min segment using the English Lexicon Project (Balota et al., 2007). Excluded words included personal names (e.g., Terry Gross and Carl Zimmer), acronyms (e.g., DNA), and the word “epigenetic” as it did not appear in the SUBTLWF_US_ corpus. Values of lexical frequency and phonological neighborhood density were log-transformed and then z-scored. There was a statistically significant correlation between lexical frequency and phonological neighborhood density (*R*^2^ = 0.37, *p* < 0.01). We checked the severity of the collinearity by calculating the Variance Inflation Factor (VIF), which came to 1.587. VIF values below five are considered to be of low concern (O’Brien, 2007). Thus, the relationship between the lexical factors is unlikely to limit our ability to accurately model word frequency and neighborhood density. The timing for each word’s onset was extracted using the software Praat (Boersma & Weenink, 2022). These were used as the stimulus onset times in the amplitude-modulated regression, which is described in detail in the fMRI Data Analysis section.

### MRI Procedure

After obtaining high-resolution structural scans, each participant was told that they were going to listen to 10 min of a podcast. The podcast audio was delivered via MRI-compatible earbuds (Avotech Silent Scan SS3300, Stuart, FL) during a single functional run. There was no visual (i.e., fixation cross) or behavioral component; participants merely listened to the podcast and were instructed to keep their eyes open and listen attentively.

### fMRI Acquisition

Structural and functional MRI images were acquired from a 3-T Prisma Scanner (Erlanger, Germany). T1-weighted structural images were acquired using a magnetization prepared rapid gradient echo (MPRAGE) sequence (repetition time [TR] = 2,400 ms, echo time [TE] = 2.22 ms, inversion time = 1,000 ms, flip angle = 8°, 300 × 320 matrix, voxel size = 0.8 × 0.8 × 0.8 mm^3^). Functional volumes were collected every 1,000 ms (TR = 1,000 ms, TE = 25 ms) in an ascending, interleaved order with an accelerated multiband sequence (multi-band factor = 4, 52 slices, 2.5-mm thick, 110 × 110 acquisition matrix, flip angle = 62°).

### fMRI Data Analysis

All analyses were completed using AFNI (Cox, 1996). Functional images were preprocessed using a standard pipeline. Images were de-obliqued, outlier volumes were censored, motion corrected via a six-parameter rigid body transform and the aligned functional images to each participant’s skull-stripped anatomical images. Initial normalization used the Talairach atlas (Talairach & Tournoux, 1988), but for the purpose of mapping to the Montreal Neurological Institute (MNI)-transformed cerebellar atlas, output of the first-level regression was then warped to MNI space. After normalization, images were smoothed using a 4-mm Gaussian kernel and all motion and signal outliers were removed following standard AFNI procedures. As the current study is exclusively concerned with patterns of activity within the cerebellum, we created individual participant masks using a native AFNI cerebellum mask to restrict the analysis of the functional data to the cerebellum. We then combined the individual masks to create a group cerebellum mask with overlapping voxels for at least 75 out of 79 participants. To do this, we first created by-participant masks (generated during AFNI pre-processing) to identify all voxels with functional data that fell within the cerebellum. These masks were aligned and overlaid, and we excluded any voxel for which there was not usable functional data for at least 75 participants. This procedure ensures that each voxel will have representation from the majority of participants, and also avoids shrinking the size of the group mask unduly if a few participants have atypical anatomy.

To assess word-level sensitivity to variation in lexical frequency and phonological neighborhood density, we used amplitude-modulated regression. For this approach, we created a single time-series vector containing the onset of each word in the podcast together with both the corresponding lexical frequency and phonological neighborhood density values. The participant-level regression using this time-series vector was implemented using the “-stim_times_AM2” flag in the *3dDeconvolve* function in AFNI. The vector was convolved with a canonical HRF function and further regressed with the six movement parameters generated during preprocessing. The output, for each participant, was two amplitude-modulated by-voxel fit coefficients—one modeling sensitivity to lexical frequency and the other to phonological neighborhood density. As this was a simultaneous regression, we were able to evaluate the cerebellar sensitivity to lexical frequency while controlling for phonological neighborhood density and vice versa.

We ran two group-level analyses, each estimating the main effects of BOLD signal changes to lexical frequency and phonological neighborhood density (separately) versus an implicit baseline (see Figure 1B and 1D). Group-level comparisons were done with a *t* test using the *3dttest++* function in AFNI. Outputs of each test were constrained to the group-level cerebellum mask described above and cluster thresholded. Cluster thresholding was determined using the *3dClustSim* function via a mixed autocorrelation function that used 10,000 Monte Carlo simulations (Cox et al., 2017) on the group mask. We estimated the group-level spatial smoothness by averaging across all participants’ noise smoothness values by extracting their ACF parameters using *3dFWHMx* and including the “-acf” flag within *3dClustSim*. This approach to thresholding fMRI data has been shown to address the concerns associated with Type I error rates in fMRI analyses (Eklund et al., 2016). The corrected group-level threshold was set at a voxelwise threshold of *p* < 0.001 with an alpha value of 0.01, used a two-sided thresholding, and a cluster size of 32 voxels.

**Figure 2.**
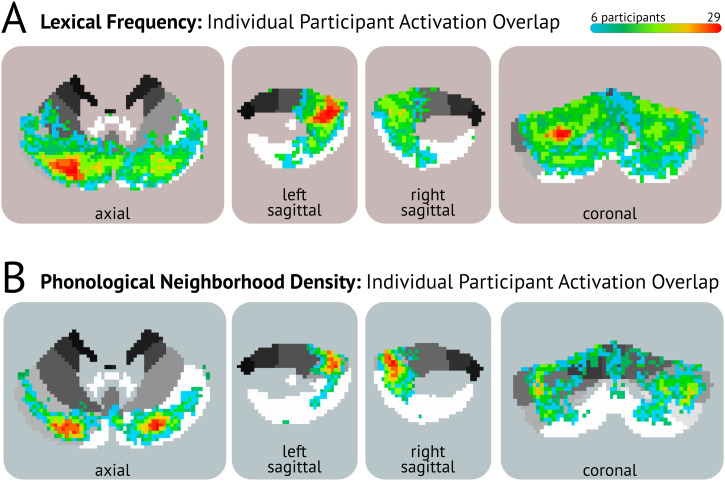
Individual-level activation overlap maps. Slice levels are identical to those in Figure 1. Individual clusters thresholded at *p* < 0.01 (voxelwise *p* < 0.01, 78 contiguous voxels) and voxels must be shared by at least six other participants. (A) Heat map showing individual-level voxel overlap for sensitivity to lexical frequency. Hotter colors indicate greater overlap, with a maximum of 29 participants activating the same voxel. (B) Heat map showing individual-level voxel overlap for sensitivity to phonological neighborhood density.

To visualize individual differences and capture variability in the localization of the effect of our regressors of interest, we constructed a heat map (limited to the bounds of the cerebellum) that layered individual activation clusters by combining the betas from the first-level regression. For individual-level activation thresholds, we set the cluster threshold to a voxelwise threshold of *p* < 0.01 at an alpha of 0.05, using two-sided thresholding, cluster size: 78 voxels. At this threshold, eight participants did not have any significant clusters for lexical frequency (Figure 2A; *n* = 71) and nine participants did not have any significant clusters for phonological neighborhood density (Figure 2B; *n* = 69). The heat maps are additionally constrained depicting only voxels that were shared by at least six participants.

## RESULTS

### Group-Level Effects of Lexical Frequency and Phonological Neighborhood Density

At the group-level, a significant effect of lexical frequency emerged in three cerebellar clusters (corrected at a threshold of *p* < 0.01; voxelwise threshold of *p* < 0.001, 32 voxel minimum cluster size). Each cluster showed a positive correlation with lexical frequency such that increased lexical frequency was associated with increased activation in these regions. As shown in Figure 1B, the two largest clusters were in right and left Crus I—extending into inferior and lateral portions of Crus II—and superior parts of Lobule VI (see Figure 1A for labeled cerebellar atlas). A small cluster was also found in Lobule IX (see Table 1).

**Table 1.** Results of group amplitude-modulated analyses

Area	Cluster size in voxels	Maximum intensity coordinates (mm)			Maximum *t* value
		*x*	*y*	*z*	
Lexical frequency					
Left Crus I/Crus II	941	−19	−75	−34	6.81
Right Crus I/Crus II	727	25	−71	−34	6.81
Lobule IX	163	5	−53	−40	3.68

Phonological neighborhood density					
Right Crus I/Crus II	463	29	−75	−34	−5.19
Left Crus I/Crus II	77	−21	−77	−34	−4.25

Phonological neighborhood density effects emerged in two bilateral clusters—located in Crus I and extended into Crus II (see Figure 1D). The size of the two clusters differed, however, showing a right-hemisphere bias with the right cluster spanning 463 voxels and the left cluster with only 77 voxels (see Table 1). Phonological neighborhood measures were negatively correlated with changes in BOLD (i.e., words with sparse neighborhoods were associated with greater activity in both clusters).

To facilitate spatial comparison of the two bilateral Crus I/Crus II clusters for each lexical factor, we computed the overlap using *3dcalc* and *3dABoverlap*—with the resulting overlap shown in Figure 1B. There was a remarkable degree of overlap. For the left cluster, the overlap encompassed an extensive area with only two voxels uniquely sensitive to phonological neighborhood density. In the right cerebellar cluster, 41 voxels were uniquely sensitive to phonological neighborhood density.

### Individual Variability

To explore potential variability in the localization of lexical sensitivity in the cerebellum, we mapped clusters sensitive to lexical frequency (see Figure 2A) and phonological neighborhood density (see Figure 2B) for each participant. Overlap across participants is shown qualitatively via a heat map, with hotter colors indicating a greater number of participants activating the same voxel. For lexical frequency, the greatest overlap was found primarily in a region corresponding to the left cerebellar Crus I/Crus II cluster, which also emerged in the group results. There was less consistency in the activation pattern in the right cerebellar hemisphere. Overall, there were widespread effects of lexical frequency. Across individuals, effects of frequency were additionally observed in Lobules V, VII, VIII, and IX. Activation associated with phonological neighborhood density was more focal across participants and landed mostly in Crus I/Crus II. For phonological neighborhood density, the hottest regions were similar to those found at the group-level. However, there seemed to be less of a right-hemisphere bias observed in the group-level results.

## DISCUSSION

The current study’s aims were twofold. First, we built on existing fMRI studies of naturalistic continuous speech processing (Brennan et al., 2016; LeBel et al., 2021). We modeled word-by-word variation in two lexical statistics, which have been pivotal in our understanding of language processing, while participants passively listened to 10 min of a popular podcast. As evident in the results, an amplitude-modulated regression approach identified variation of the BOLD signal in the cerebellum associated with these lexical properties during continuous listening. Our second aim was concerned with the nature of the cerebellum’s response to variation in lexical frequency and phonological neighborhood density. At the group level, functional sensitivity to both lexical frequency and phonological neighborhood density fell in predicted subregions—primarily bilateral Crus I and Crus II, extending somewhat into Lobule IX. At the individual level, there was a considerable amount of variability in the location and degree of activation to both factors.

There was a positive relationship between activation and lexical frequency and a negative relationship between activation and phonological neighborhood density. The correlations between cerebellar activity and each lexical measure were in a consistent direction, suggesting that the cerebellum is sensitive to lexical-level properties that are known to affect word recognition. Of further interest is whether sensitivity to phonological neighborhood density and lexical frequency in Crus I/Crus II emerged in common voxels. Figure 1 shows the extent of the overlap; the majority of the voxels sensitive to phonological neighborhood density were shared with those activated for frequency effects. Since both factors are known to impact lexical processing, this naturally leads to the question: What role might the cerebellum play in online language comprehension?

When listening to spoken language, the statistical distributions of the incoming speech signal are tracked at multiple levels of processing (e.g., Luthra et al., 2021; Theodore & Miller, 2008). However, much of what we know about the neural responses associated with these distributions has almost exclusively been restricted to the cerebral cortex. With a growing consensus that the cerebellum is integral to the neural architecture that supports spoken language comprehension (Bonhage et al., 2015; Geva et al., 2021; Moberget & Ivry, 2016; Stoodley & Schmahmann, 2009), we focus on how the cerebellum might rely on internal models (Ito, 2005; Ivry, 1997; Ivry & Keele, 1989; Wolpert et al., 1998) that represent word-sound relationships. The cerebellum has been proposed to generate perceptual and linguistic predictions, and also to integrate single words with the broader linguistic context, by “tracking” word-level behaviorally relevant distributions.

The work on the cerebellum and language-based internal models has focused primarily on the role of the cerebellum in generating linguistic predictions; for example, investigating the cerebellum’s role in semantically generated lexical predictions from a preceding sentence context (Moberget et al., 2014). While there is strong evidence for cerebellar involvement in this process, there is debate regarding the nature of these linguistic predictions; whether they are generated from motor processes (Pickering & Gambi, 2018), involve associative semantic relationships (e.g., Argyropoulos, 2011), or occur at multiple levels (Skipper & Lametti, 2021). Our finding of a common area that is sensitive to both lexical frequency and phonological neighborhood density suggests that phonological-lexical statistics are tracked by the cerebellum and may help in generating predictions and/or encoding prediction errors at multiple levels of processing. Importantly, the clusters associated with lexical variation were located in Crus I/Crus II. These subregions are not associated with motor processing (Stoodley et al., 2012), suggesting that the predictions generated during listening and comprehension are not limited to motor-based processes.

The current study used a natural, continuous speech stimulus. Continuous speech contains rich context cues, at the sentential and the conversational level, motivating a more active listening experience that is conducive for generating predictions about upcoming linguistic elements and for integrating words into the broader context. There is little dispute that predictions are beneficial when deciphering ambiguous speech and listening in noisy environments (for review, see Mattys et al., 2012). In noise, prediction accuracy is modulated by lexical frequency; high frequency words are more easily and accurately predicted than low frequency words (Benkı´, 2003; Besner & Young, 2022; Huizeling et al., 2022). Words with denser phonological neighborhoods are likely to generate a greater number of potential candidates (or predictions) compatible at different time points of the unfolding speech signal (Luce & Pisoni, 1998). Thus, our findings are broadly consistent with a prediction interpretation in which lexical knowledge contributes to predictions about what we hear. In particular, the voxels sensitive to both frequency and neighborhood density in Crus I/Crus II may be due to their involvement in lexically generated phonological predictions, as has been previously suggested (Guediche et al., 2014; Guediche et al., 2015; Lesage et al., 2017).

Prediction is only one possible framework for interpreting the cerebellum’s role in speech comprehension that fits with the current results. For instance, semantic integration of a given word with surrounding words—and the entire discourse—is also affected by the word’s properties. Evidence points to higher frequency words being processed faster than lower frequency words (reviewed in Huizeling et al., 2022). If higher frequency words are accessed more easily and more rapidly, we might expect that these same words are easier to integrate with previously heard context (Ferreira & Chantavarin, 2018; Roodenrys et al., 2002). Further, frequency effects tap into retrieval and integration mechanisms at multiple levels of processing including the word’s phonological form (Fox, 1984) and discourse-level context (Brodbeck et al., 2022). Phonological neighborhood density may also affect word recognition as it unfolds over time. Since the relatedness of a preceding semantic context is also known to modulate the magnitude of neighborhood density effects (Chen & Mirman, 2015), phonological neighborhood density may also impact ease of contextual semantic integration.

It may be that the cerebellum reflects the ease of processing individual words as modulated solely by a given word’s lexical properties (either in terms of prediction, integration, or both); an interpretation that cannot be disentangled from the ease of processing words that vary in their demands on domain-general resources (e.g., working memory demands). To this point, the Crus I/Crus II subregions that emerged in the current study are also implicated in domain-general processes via functional connectivity with cortex. Resting state functional connectivity studies have shown that portions of Crus I/Crus II are associated with the default mode network (Buckner et al., 2011; Xue et al., 2021) as well as fronto-parietal executive control networks (Habas, 2021; Habas et al., 2009). Resting state approaches, however, speak to whether certain regions pattern together at rest. Thus, they are limited in their ability to speak to task-specific connectivity. Studies that have used task-based functional connectivity analyses for language processes tend to show Crus I/Crus II patterning with the canonical cortical language network (Ashida et al., 2019; King et al., 2023; Xue et al., 2021). There does appear to be some overlap between language-specific and more domain-general functional networks in cognitive cerebellar regions (Ashida et al., 2019), and there is growing evidence that domain-general networks might contribute to various aspects of language processing. Concerning the default mode network specifically, the traditional view was that it supported task disengagement functions such as mind-wandering (for review, see Raichle, 2015). More recently, the default mode network is shown to have potential involvement in language-related tasks such as narrative comprehension and semantic access (Chang et al., 2022; Simony et al., 2016), as well as direct overlap with the semantic network (Binder et al., 2009). Interestingly, the default mode network (including the cerebellum) can be subdivided into multiple networks, some of which are implicated in language processing (Buckner & DiNicola, 2019; Gordon et al., 2020). Regardless of whether sensitivity to word-level information in the cerebellum is due to domain-general or language-specific mechanisms, there is mounting evidence pointing toward the cerebellum’s involvement in language processing (Schmahmann, 2019) that cannot be discounted. Differences in connectivity findings may indicate that traditional boundaries between online language processing and domain-general functions are rather artificial (Binder et al., 2009; Dohmatob et al., 2020). Further work is needed to carefully parcellate the cortical functional networks in specific subregions of the cerebellum across a wide range of tasks including continuous listening.

On the issue of specificity, group-level effects do not necessarily capture what emerges at the individual level, especially if there is some degree of functional and/or structural variability (e.g., Okada & Hickok, 2006). In the current study, we observed a considerable amount of overlap at the individual level in location and degree of activation to both factors, and also variation across individuals (see Figure 2). Individual activation maps indicate that articulatory (Lobule V), perceptual (Lobule VI), and other cognitive processes (Lobules VII, VIII, and IX) might also contribute to the emergence of lexical frequency effects in the cerebellum. Individual variation in sensitivity to neighborhood density was not as widespread as that of lexical frequency nor was it as variable across individuals. Density effects were more restricted to Crus I/Crus II and to closely surrounding areas and was more right lateralized. Since cerebellar connections to cerebral cortex are contralateral, the right-lateralized effects of phonological neighborhood density in the cerebellum are consistent with the involvement of the more dominant left-hemisphere language network (Murdoch, 2010). These findings suggest a potential cascading influence of frequency and neighborhood density across the language system that varies across individuals. Despite this variability, Crus I/Crus II remained the most probable sites of activation across participants for both factors, supporting the group level results. Visualizing the individual-level data helps to confirm the group-level results, lending confidence to the patterns and functional loci of activity in the cerebellum reported in the current study.

## CONCLUSION

We add to mounting evidence that calls for including the cerebellum (especially Crus I/Crus II) into neuroanatomical accounts of language processing as a critical part of the underlying functional architecture, and for investigating the cerebellum’s role during language processing more broadly. While our findings cannot identify the specific cerebellar mechanisms involved in spoken language processing, as they are consistent with many possible interpretations, they do highlight the cerebellum’s role in word-level processing. Importantly, future work can further tease apart the contributions of specific subregions in different aspects of language processing by using targeted manipulations of stimulus features, analyses methods (including connectivity), and other methodological tools that improve localization precision and accuracy.

## ACKNOWLEDGMENTS

We thank our participants for giving us their time and effort. We also thank the many undergraduate research assistants for their dedication to the project.

## FUNDING INFORMATION

Christopher C. Heffner, National Science Foundation (https://dx.doi.org/10.13039/100000001), Award ID: SMA 1714858. Emily B. Myers, National Institutes of Health (https://dx.doi.org/10.13039/100000002), Award ID: R01 DC013064.

## AUTHOR CONTRIBUTIONS

**Hannah Mechtenberg**: Data curation, Formal analysis, Writing – original draft, Writing – review & editing. **Christopher C. Heffner**: Funding acquisition, Writing – review & editing. **Emily B. Myers**: Funding acquisition, Writing – review & editing. **Sara Guediche**: Writing – original draft, Writing – review & editing.

## CODE AND DATA AVAILABILITY STATEMENT

The data and code that support the findings of this study are openly available on GitHub at https://github.com/Hannah-r-Mechtenberg/cerebellum-podcast-fmri. We are sharing the downstream statistical data files that directly support the current study. We have provided the scripts that these statistical files were derived from (including the regression script and associated timing file), as well as the group-level statistics script and the script to compute functional cluster overlap.

The raw, whole-brain data files (structural and functional) will be available upon request while our group is currently working on several lines of research drawing from these data. Pending publication of these in-progress projects, we will update the repository with whole brain files as well as any additional files or scripts requested by interested parties.

## TECHNICAL TERMS

Lexical frequency: Indicates the commonness of a word, with words that are more commonly encountered having higher frequency values.
Phonological neighborhood density: Indicates the number of words that differ from a target word by one phoneme, with higher values describing words with more neighbors (e.g., cat).
Internal models: Context-specific representations that allow for efficient processing of incoming sensory and motor information.
Amplitude modulated regression: In fMRI, an analysis technique that models how changes in neural activity correlate with changes in a continuous variable of interest.
